# Immunopathogenesis of Hepatic Brucellosis

**DOI:** 10.3389/fcimb.2019.00423

**Published:** 2019-12-20

**Authors:** Guillermo Hernán Giambartolomei, María Victoria Delpino

**Affiliations:** Instituto de Inmunología, Genética y Metabolismo (INIGEM), Universidad de Buenos Aires, Buenos Aires (UBA), Consejo Nacional de Investigaciones Científicas y Técnicas (CONICET), Buenos Aires, Argentina

**Keywords:** liver, fibrosis, inflamación, hepatocite, stellate cells (Ito cells)

## Abstract

The hepatic immune system can induce rapid and controlled responses to pathogenic microorganisms and tumor cells. Accordingly, most of the microorganisms that reach the liver through the blood are eliminated. However, some of them, including *Brucella* spp., take advantage of the immunotolerant capacity of the liver to persist in the host. *Brucella* has a predilection for surviving in the reticuloendothelial system, with the liver being the largest organ of this system in the human body. Therefore, its involvement in brucellosis is practically invariable. In patients with active brucellosis, the liver is commonly affected, and the most frequent clinical manifestation is hepatosplenomegaly. The molecular mechanisms implicated in liver damage have been recently elucidated. It has been demonstrated how *Brucella* interacts with hepatocytes inducing its death by apoptosis. The inflammatory microenvironment and the direct effect of *Brucella* on hepatic stellate cells (HSC) induce their activation and turn these cells from its quiescent form to their fibrogenic phenotype. This HSC activation induced by *Brucella* infection relies on the presence of a functional type IV secretion system and the effector protein BPE005 through a mechanism involved in the activation of the autophagic pathway. Finally, the molecular mechanisms of liver brucellosis observed so far are shedding light on how the interaction of *Brucella* with liver cells may play an important role in the discovery of new targets to control the infection. In this review, we report the current understanding of the interaction between liver structural cells and immune system cells during *Brucella* infection.

## Brucellosis

Brucellosis is a common zoonotic disease distributed around the world. The disease is rare in industrialized countries because of the routine screening of domestic livestock and animal vaccination program (Pappas et al., 2006; Mancini et al., 2014). Recently, case studies and review of published cases have been carried out to determine the prevalence of brucellosis in endemic regions. However, it should be borne in mind that because many areas with endemic brucellosis have poor infrastructure, it is likely that the incidence of the disease is underestimated. There are a reported 500,000 incident cases of human brucellosis per year. However, true incidence is estimated to be 5,000,000 to 12,500,000 cases annually (Godfroid et al., 2013; Hull and Schumaker, 2018). The clinical disease is common in the Middle East, Asia, Africa, South and Central America, the Mediterranean Basin, and the Caribbean (Pappas et al., 2006; Mancini et al., 2014; Cross et al., 2019).

At present, 12 species of *Brucella* genus have been described, each of which has its natural host, including domestic and farm animals, as well as wild animals, such as camels, bison, foxes, cetaceans, among others. *Brucella* is an expanding genus, and the most recent species were isolated from amphibians (Eisenberg et al., 2012; Fischer et al., 2012). The bacteria are transmitted from animals to human by ingestion of infected food products (meat or raw milk), direct contact with infected animals or their tissues, or inhalation of aerosols. Humans are accidental hosts, but brucellosis continues to be a major public health concern worldwide and is the most widespread zoonotic infection.

*Brucella* genus does not exhibit classic virulence factors, such as exotoxins, exoproteases, cytolysins, or other exoenzymes (Moreno and Moriyon, 2002). Observed tissue harm is a result of inflammatory immune responses through the activation of host immune responses after recognition of brucellar antigens by immune receptors, such as Toll-like receptors (TLR) and inflammasomes (Campos et al., 2004, 2017; Giambartolomei et al., 2004; Zwerdling et al., 2008, 2009; García Samartino et al., 2010; de Almeida et al., 2011, 2013; Delpino et al., 2012; Gomes et al., 2013). Notwithstanding, with its intracellular lifestyle, *Brucella* limits exposure to innate and adaptive immune responses and leads the clinical manifestations of the disease and pathology. *Brucella* takes advantage of intracellular destruction by restringing fusion of *Brucella*-containing vacuoles with lysosomal compartments in a mechanism mediated by the type IV secretion system (T4SS) (Comerci et al., 2001). Also, *Brucella* inhibits the apoptosis of infected macrophages and prevents the development of adequate adaptive immune response by the inhibition of antigen presentation (de Figueiredo et al., 2015; Barrionuevo and Giambartolomei, 2019).

## Clinical Features of *Brucella* Liver Infection

The liver is the most commonly affected organ in patients with active brucellosis. Accordingly, clinical and biochemical records of liver involvement have been observed in up to 50% of patients with active disease (Colmenero et al., 1996). Histopathological analyses of liver biopsies from a large number of patients with brucellosis have revealed liver parenchyma lesions due to inflammation, including focal areas of cellular inflammation with minimal necrosis of liver cells or the presence of granulomas with different localizations in parenchymal tissue and portal space (Akritidis et al., 2007). The pathology report on liver granulomas in *Brucella* infection usually shows necrotizing granulomas with a peripheral halo of epithelioid cells, lymphocytes, and plasma cells, as well as polymorphonuclear infiltrate in the necrotic area (Colmenero Jde et al., 2002; Villar et al., 2002). There are differences in the histological evaluation of liver manifestations in human brucellosis due to several causes. One of them is that most reports are retrospective and lack bacteriological confirmation. Also, previous reports with bacteriological confirmation did not always specify the species of *Brucella* involved. Moreover, not all of them used the same criteria to define granuloma (for example, presence of epithelioid cells or presence of giant cells) (Adams, 1976).

Liver biopsies also presented evidence of hepatitis (Akritidis et al., 2007). The liver conducts the metabolism of carbohydrates, proteins, and fats. Some of the enzymes and the end products of these metabolic pathways may be utilized as biochemical markers of liver dysfunction because they are very sensitive to any abnormality that takes place. Despite the abovementioned, the liver function is frequently normal in *Brucella*-infected individuals. The total serum bilirubin may be slightly increased, and the total serum protein, albumin, and globulin are usually normal. The most frequent abnormalities are shown by the increase in transaminases and alkaline phosphatase, although they are non-specific (Young, 1995; Madkour, 2001). In these patients, the presence of inflammatory infiltrates is also common, and most of them present parenchymal necrosis (Madkour, 2001; Akritidis et al., 2007).

## The Liver as an Immune Organ

The liver is usually regarded only as a non-immunological organ involved mainly in metabolic, nutrient storage, and detoxification activities. However, it is a member of the immune system responsible for the production of acute-phase proteins, chemokines, cytokines, complement proteins, and it also carries diverse populations of resident immune cells (O'Farrelly and Crispe, 1999; Crispe, 2009; Nemeth et al., 2009). Notwithstanding, the spleen is the main critical mediator in the clearance of blood pathogens, which overlaps with the function of the liver (Robinson et al., 2016).

Lymphocyte populations are present in the parenchyma and the portal tracts of the liver. These populations consist of conventional and unconventional lymphocytes of innate immunity, NKT and NK cells, as well as cells of the adaptive immune system, T and B cells (Freitas-Lopes et al., 2017). Resident antigen-presenting cells are also abundant in the liver. These cells are able to capture antigens that enter through the liver or are released by dead or infected hepatocytes. The group of resident antigen-presenting cells comprises Kupffer cells (members of the reticuloendothelial system) (Gale et al., 1978), liver sinusoidal endothelial cells (LSEC) (a particular type of vascular endothelial cells) (Steffan et al., 1986), and dendritic cells (DC) (Prickett et al., 1988; Lau and Thomson, 2003). The presence of these cells is essential for the maintenance of liver tolerance in physiological conditions (Robinson et al., 2016).

Kupffer cells are the liver resident macrophages that adhere to sinusoidal endothelial cells inside the sinusoids (Smith, 2013). This localization is adequate to perform its function as a scavenger removing protein complexes, small particles, senescent red blood cells, and cell debris from the portal blood via pattern recognition receptors (PRRs) (Petrasek et al., 2012).

LSEC are specialized endothelial cells that by the absence of diaphragm and lack of basement membrane are the most permeable endothelial cells in the body. In physiological conditions, these cells maintain hepatic stellate cell (HSC) quiescence inhibiting fibrosis development. These cells have a high phagocytic capacity and the molecules that promote antigen processing and presentation with efficacy similar to that of DC (Steffan et al., 1986; Lohse et al., 1996; Knolle et al., 1999).

Resident hepatic DC are located around the central veins and portal tracts. The presence of interleukin (IL)-10 and transforming growth factor (TGF)-β secreted by Kupffer and LSEC cells in the absence of infection provides a microenvironment capable of generating tolerant resident DC. The activation of these cells increases their capacity to migrate via the space of Disse to the lymphatic vessels in the portal tracts and then to the extrahepatic lymph nodes (Matsuno et al., 1996; Kudo et al., 1997).

Additionally, HSC and hepatocytes contribute to the immune homeostasis of the liver. Quiescent HSC exert immunoregulatory roles secreting chemokines, chemokine receptors, macrophage inflammatory proteins (MIPs), and TLR and also function as antigen-presenting cells (Friedman, 2008; Hernandez-Gea and Friedman, 2011). Hepatocytes can also participate as antigen-presenting cells, but they do not express the costimulatory molecules CD80 and CD86 (Bertolino et al., 1998). Therefore, hepatocytes induce T-cell functional activation but fail to promote survival (Bertolino et al., 1998). In addition, hepatocytes can also express PD-L1 in response to interferon (IFN) of type I and II with the consequent induction of T-cell apoptosis contributing to liver tolerance (Mühlbauer et al., 2006). This immunotolerant capacity of the liver is due to its structure, with resident immune cells in constant stimulation and the hepatic blood supply that creates a unique cytokine and growth factor milieu. This microenvironment determines the balance between tolerance and inflammation in the healthy liver. The complex of cytokine milieu in adult liver, in the absence of pathological inflammation, includes basal expression of proinflammatory cytokines IL-15, IL-7, IL-2, IL-12, IFN-γ, and the anti-inflammatory cytokines IL-10, IL-13, and TFG-β (Golden-Mason et al., 2004; Kelly et al., 2006). The tolerogenic environment is maintained by regulatory myeloid populations, such as myeloid-derived suppressor cells (MDSC), that mediate their suppressive activity through the production of IL-10 and TGF-β (Gabrilovich and Nagaraj, 2009). The transmigration of monocytes leads to MDSC differentiation and activation, contributing to the immunoregulatory capacity of the liver (Sander et al., 2010; Zimmermann et al., 2015). In particular, the impact of IL-10 in *Brucella* persistence and establishment of chronic infection through the modulation of macrophages has been demonstrated previously by using IL-10-deficient mice (Xavier et al., 2013).

Nevertheless, this immune tolerance, the hepatic immune system is able to induce rapid and controlled responses to pathogenic microorganisms and tumor cells (Robinson et al., 2016). Accordingly, most of the microorganisms that reach the liver through the blood are eliminated. Although the liver has several mechanisms to resist and to eliminate infectious agents, some of them, such as *Brucella* spp, take advantage of the immunotolerant capacity of the liver to escape from the immune response and persist in the host. Besides, because the liver is the organ with the largest number of resident macrophages (Heymann and Tacke, 2016), we can speculate that the liver constitutes a place for *Brucella* persistence.

## *Brucella* and HSC in Fibrosis

HSC reside in the liver between the hepatocytes and the small blood vessels. These cells are characterized as containing intracellular lipid droplets and protrusions that extend around the blood vessels. During liver damage, these cells are activated and secrete collagen with the formation of scar tissue, leading to chronic fibrosis or cirrhosis (Xu et al., 2012).

*Brucella* is an infectious stimulus that causes HSC activation that involves the conversion of quiescent cells into myofibroblasts, as revealed by the increase in α-smooth muscle actin (SMA) expression, the increase in collagen secretion, the inhibition of matrix metalloproteinase (MMP)-9 secretion, the induction of the tissue inhibitor of metalloproteinases (TIMP)-1, and the secretion of the master regulator of fibrosis TGF-β (Arriola Benitez et al., 2013). TGF-β was identified as a main driver of HSC activation, and several approaches targeting the TGF-β signaling pathway were successfully used to engage fibrosis in animal models of chronic liver diseases (Dooley and ten Dijke, 2012; Puche et al., 2013).

HSC secrete the chemokines IL-8 and the monocyte chemotactic protein (MCP)-1 in response to *Brucella abortus* infection that could attract to the site of infection, neutrophils and monocytes, respectively (Arriola Benitez et al., 2013). It has been demonstrated that *B. abortus*-infected monocytes could not reverse the fibrotic phenotype in *B. abortus*-infected HSC. However, *B. abortus*-infected monocytes inhibit collagen deposition and induce MMP-9 secretion in uninfected HSC (Arriola Benitez et al., 2013). This may explain, at least in part, why many patients with brucellosis have an inflammatory infiltrate, but only some of them develop cirrhosis. Moreover, in patients with brucellosis and cirrhosis, other causes such as viral hepatitis or alcoholic cirrhosis, have not been ruled out (Madkour, 2001).

**Figure 1:**
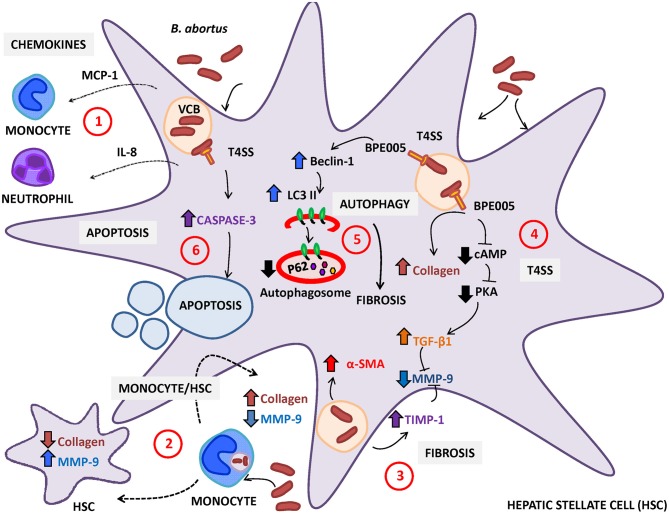
*Brucella abortus* infection modulates the activation of hepatic stellate cells (HSC). (1) HSC secrete monocyte chemotactic protein (MCP)-1 and interleukin (IL)-8 (chemoattractant of monocytes and neutrophils, respectively) in response to *B. abortus* infection. (2) Resident macrophages or monocytes attracted to the site of infection could be infected by *B. abortus* and then modulate the expression of collagen and MMP-9 depending on the status of HSC (infected or not). In non-infected HSC, *B. abortus*-infected monocytes induce an inflammatory phenotype characterized by the increase in MMP-9 expression and the inhibition of collagen deposition. In contrast, *B. abortus*-infected monocytes induce the inhibition of matrix metalloproteinase (MMP)-9 secretion and the increase in collagen deposition in infected HSC. (3) *B. abortus* infection of HSC induces HSC activation by inducing a fibrotic phenotype characterized by the expression of α-smooth muscle actin (α-SMA), collagen deposition, and inhibition of MMP-9 through a mechanism that is dependent on the increase in transforming growth factor (TGF)-β and tissue inhibitor of metalloproteinase (TIMP)-1. (4) Fibrosis induction by *B. abortus* infection was dependent on the Type IV secretion system (T4SS) and its secreted effector BPE005 in a mechanism that depends on the cyclic AMP (cAMP)/protein kinase A (PKA) signaling pathway. (5) Fibrosis induction is also dependent on autophagy pathway induction characterized by the increase in the expression of LC3II and Beclin-1 and the inhibition of p62 expression. (6) Then, *B. abortus* infection induces cell death by apoptosis of activated HSC in a mechanism that is dependent on a functional T4SS and caspase 3 cleavage.

Until now, it is unclear that *Brucella* infection can directly or indirectly modulate HSC activity and, consequently, the deposition of extracellular matrix. As in other liver diseases, HSC activation during *B. abortus* infection could contribute to granuloma formation by laying down a ring of collagen to encapsulate the granuloma (Chuah et al., 2014) (Figure 1).

## *Brucella* Type IV Secretion System in Fibrosis

T4SSs are a multiprotein complex involved in the translocation of nucleoproteins and/or protein substrates across the bacterial cell envelope to the host cell (Zechner et al., 2012). In *Brucella*, T4SS is encoded by the *virB* operon, which consists of 12 genes (virB1-12) located on chromosome II. The transcription of the *virB* operon is controlled by the promoter upstream of *virB*1 (O'Callaghan et al., 1999; Sieira et al., 2000). T4SS protein substrates have been shown to modulate several cellular processes in the host cell, such as apoptosis, vesicle trafficking, ubiquitination, and so on (Ninio and Roy, 2007; Franco et al., 2009). In *Brucella*, T4SS has been shown to be involved in the modulation of the immune system during infection (Roux et al., 2007; Rolán and Tsolis, 2008; Gomes et al., 2013); *Brucella* protein effector (BPE)005 is particularly involved in liver fibrosis (Arriola Benitez et al., 2016). The predicted structure BPE005 suggests that it might have an effect on cyclic AMP (cAMP) signaling pathways (Marchesini et al., 2011). In the HSC, BPE005 may mediate its effect by blocking the binding between cAMP and protein kinase A (PKA) with the concomitant fibrosis induction, as demonstrated in studies performed *in vitro* (Arriola Benitez et al., 2016). Interestingly, the role of BPE005 in the modulation of the fibrotic phenotype during *B. abortus* infection was confirmed in an *in vivo* model in mice infected with a *B. abortus* BPE005 mutant. The histological analysis by Masson's trichrome staining revealed that the level of fibrotic patches is lower in mice infected with BPE005 mutants than in those infected with the wild-type counterpart. Accordingly, levels of collagen and TGF-β were lower in mice infected with the *B. abortus* BPE005 mutant (Arriola Benitez et al., 2016).

The role of the cAMP/PKA signaling pathway in the liver has been reported previously. The involvement of this pathway was demonstrated in various metabolic functions with the main effect in the facilitation of carbohydrate and lipid metabolism. cAMP plays a major role in the modulation of the HSC function by inhibiting profibrogenic pathways in HSC (Lopez-Sanchez et al., 2014).

Autophagy is involved in the fibrotic response during chronic hepatic lesion caused by hepatitis virus infection, alcohol abuse, and nonalcoholic steatohepatitis (Song et al., 2014).

Autophagy is a catabolic intracellular pathway that targets defective or excessive organelles to the lysosomes for degradation into amino acids, free fatty acids, or other small molecules used for material recycling or energy harvesting (Mao and Fan, 2015). Autophagy, usually stimulated by energy restriction, stress, or inflammation, is regarded as a survival mechanism that plays a critical role in maintaining cellular homeostasis, which is involved in many human disorders, such as fibrotic disease (Yin et al., 2008). In fibrosis, autophagy is mostly a cell survival mechanism that attenuates hepatic inflammatory injury and ultimately induces liver fibrosis (Mao and Fan, 2015). In addition, and supporting the role of BPE005 in the induction of HSC activation to a fibrotic phenotype, it has been demonstrated that autophagy is involved in the fibrotic response due to *B. abortus*, which depends on a functional T4SS and its effector BPE005 (Arriola Benitez et al., 2018). Autophagy was revealed by the upregulation of the LC3II/LC3I ratio and Beclin-1 expression and by the inhibition of p62 expression in infected cells (Arriola Benitez et al., 2018). However, further studies are necessary to determine if BPE005 could be a therapeutic target because this factor induces a fibrogenic phenotype that could help not only the host in the response to the inflammatory damage but also in the formation of granulomas likely favoring the persistence of bacterium.

Of note, the presence of liver cirrhosis during hepatic brucellosis is a debatable issue (Madkour, 2001). In addition, in murine models, cirrhosis was not observed upon infection with *B. abortus* (Arriola Benitez et al., 2013). In line with previous observations, *B. abortus* infection induces the clearance of activated HSC by apoptosis through a mechanism that is dependent on a functional T4SS and involved caspase 3 cleavage, triggering the recovery of liver fibrosis (Arriola Benitez et al., 2018) (Figure 1).

## *Brucella* and Hepatocytes

Hepatocytes are the main cells of the liver parenchyma tissue and occupy around 70–85% of the liver volume (Kmieć, 2001). Hepatocytes are known to be involved in the synthesis of proteins, glycoproteins, cholesterol, bile salts, and phospholipids. On the other hand, they contribute to the detoxification and excretion of substances. However, these cells also participate in the immune response against pathogens. Hepatocytes respond to viral, bacterial, and parasitic infections by secreting pro-inflammatory cytokines and chemokines as mentioned elsewhere (Santos et al., 2005; McCord et al., 2006; Costa et al., 2008; Heydtmann and Adams, 2009), and *B. abortus* is not the exception. *B. abortus* infects hepatocytes and induces the secretion of IL-8, the main chemoattractant for neutrophil (Delpino et al., 2010), which correlates with the neutrophil infiltration observed in the liver of *Brucella*-infected patients (Hunt and Bothwell, 1967; Colmenero Jde et al., 2002). *B. abortus* infection also induces intercellular adhesion molecule (ICAM-1) and MMP-9 secretion by hepatocytes (Delpino et al., 2010). These molecules ensure the influx of neutrophils to the tissues. ICAM-1 could facilitate the interaction of hepatocytes with neutrophils, and MMP-9 promotes neutrophil transmigration through degradation of extracellular matrix. These neutrophils could be also infected by *B. abortus*, and in response to this infection, they can contribute to the inflammatory reaction through the secretion of IL-8 and MMP-9 by inducing the expression of ICAM-1 by hepatocytes (Delpino et al., 2010).

Presence of liver parenchymal necrosis has been demonstrated in patients with hepatic manifestations of brucellosis (Akritidis et al., 2007), and hepatocytes occupy most of the parenchymal tissue. The analysis of the effects of *B. abortus* infection on hepatocyte viability revealed the induction of cell death by apoptosis and cytotoxic release of lactate dehydrogenase (LDH) in a T4SS-dependent manner (Delpino et al., 2010). Neutrophils also contribute to the induction of apoptosis and LDH release by hepatocytes as revealed by the stimulation with culture supernatants from *B. abortus*-infected neutrophils (Delpino et al., 2010).

**Figure 2:**
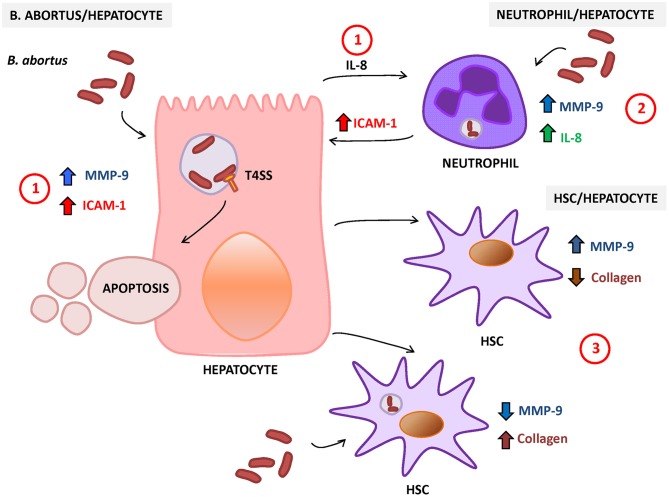
Response of hepatocytes to *Brucella abortus* infection. (1) *B. abortus* infection of hepatocyte induces the secretion of matrix metalloproteinase (MMP)-9 and interleukin (IL)-8 (chemoattractant of neutrophils) and increases the surface expression of intercellular adhesion molecule (ICAM)-1 and cell death by apoptosis. (2) *B. abortus*-infected neutrophils secrete IL-8 and MMP-9 and induce the expression of ICAM-1 on hepatocytes. (3) HSC also interact with hepatocytes, and its response is dependent on the status of HSC (infected or not). In non-infected HSC, *B. abortus*-infected hepatocytes induce an inflammatory phenotype characterized by the increase in MMP-9 expression and the inhibition of collagen deposition. In contrast, *B. abortus*-infected hepatocytes induce the inhibition of MMP-9 secretion and the increase in collagen deposition in infected HSC.

*B. abortus-*infected hepatocytes can also modulate HSC function. Supernatants from *B. abortus*-infected hepatocytes induce MMP-9 secretion and inhibit collagen deposition in LX-2 cells (Arriola Benitez et al., 2013). However, when stimulation was performed on *B. abortus*-infected HSC, supernatants from *B. abortus*-infected hepatocytes were unable to reverse the inhibitory effect of *B. abortus* infection in the inhibition of MMP-9 secretion and the induction of collagen deposition on HSC (Arriola Benitez et al., 2013). The status of HSC (infected or not) defines if *B. abortus*-infected hepatocytes induce a fibrotic or an inflammatory phenotype in these cells (Figure 2).

## *In vivo* Studies for Liver Brucellosis

The absence of a suitable animal model that can reproduce, after experimental infection, the variety of disease manifestations of human brucellosis has determined the slow progress in most of the pathobiology of focal forms of the disease. However, while laboratory rodents do not mimic all of the spectrum of clinical signs and symptoms of human disease, there are focal manifestations of liver brucellosis (Olsen and Palmer, 2014). This allows characterizing at least in part, the pathophysiological and immune manifestations of hepatic brucellosis.

Studies performed in mice infected with *Brucella melitensis* stated that most of the infected cells in the liver expressed F4/80 myeloid marker. In the peak of infection, granuloma formation occurs. Granulomas are mainly composed by CD11b+ F4/80+ MHC-II+ cells expressing iNOS/NOS2 enzyme (Copin et al., 2012). A population of these cells also expressed CD11c marker, indicating the presence of inflammatory DC. However, further analyses are necessary to determine if *Brucella* will remain in these cells or will migrate to others that protect them from the immune response (Copin et al., 2012). The delay in conducting these studies is due to the fact that the murine model does not represent all of the clinical manifestations of the disease, and that the liver biopsy is not a medical practice for the diagnosis of brucellosis.

*In vivo* studies also revealed that IL-10 production by CD25^+^CD4^+^ T cells that modulate macrophage function enhances bacterial survival and persistence in liver through the modulation of the balance between pro-inflammatory and anti-inflammatory cytokines (Xavier et al., 2013). In addition, liver histopatology from infected IL-10^−^/^−^ mice at 3 weeks revealed multifocal granulomas and liver necrosis, as occurs in wild-type mice. However, at 6 weeks postinfection, the number of granulomas was reduced in IL-10^−^/^−^ mice with respect to wild-type controls. This reduction in liver pathology was accompanied by the increase of CD4^+^CD25^+^ foxp3^+^ T cells with the expression of TGF-β (Corsetti et al., 2013). We can speculate that this reduction in liver pathology despite the increase in TGF-β can be attributed to the reduction of bacterial number and the consequent reduction in liver damage. However, further studies would be necessary to determine if the TGF-β levels produced are capable of causing liver fibrosis in these mice.

In addition, intraperitoneal infection of mice with *B. abortus* allows corroborating *in vitro* findings, among them the role of BPE005 in the induction of liver fibrosis revealed by the increase in collagen deposition in liver and the expression of TGF-β in HSC, as well as the presence of lymphocytic inflammatory infiltrate (Arriola Benitez et al., 2013, 2016).

Despite the limitations of the rodent model, it allowed to corroborate the most relevant *in vitro* findings related to human clinical outcomes.

## Concluding Remarks

Liver involvement in active brucellosis has ranged from 5 to 52% or more (Madkour, 2001). While the clinical and imaging aspect of hepatic brucellosis has been widely described, the molecular and cellular mechanisms involved in the damage and the immune response in the liver have only been partially clarified over the past 10 years. The findings summarized in this review attempt to answer the key questions about the soluble mediators, local and infiltrating cells involved in inflammatory damage during liver brucellosis.

The infection through the T4SS effector, BPE005, could modulate HSC activation to control the fibrotic process. It is likely that BPE005 could participate in granuloma formation that might act as a reservoir of bacteria contributing to the disease chronicity. Additionally, and in line with the presence of liver parenchymal necrosis observed in patients (Akritidis et al., 2007), *B. abortus* induces cell death of hepatocytes in a mechanism that also depends on a functional T4SS (Arriola Benitez et al., 2018). Altogether, this knowledge could frame the first approach toward the discovery of a new therapeutic target leading to new treatments that could be coadministrated with antibiotics aimed at reducing liver damage.

## Author Contributions

GG helped draft and correct the manuscript. MD drafted the manuscript.

### Conflict of Interest

The authors declare that the research was conducted in the absence of any commercial or financial relationships that could be construed as a potential conflict of interest.

## References

[B1] Adams D. O. (1976). The granulomatous inflammatory response. A review. Am. J. Pathol. 84, 164–192. 937513 PMC2032357

[B2] Akritidis N. Tzivras M. Delladetsima I. Stefanaki S. Moutsopoulos H. M. Pappas G. (2007). The liver in brucellosis. Clin. Gastroenterol. Hepatol. 5, 1109–1112. 10.1016/j.cgh.2006.08.010 17482524

[B3] Arriola Benitez P. C. Pesce Viglietti A. I. Herrmann C. K. Dennis V. A. Comerci D. J. Giambartolomei G. H.. (2018). *Brucella abortus* promotes a fibrotic phenotype in hepatic stellate cells, with concomitant activation of the autophagy pathway. Infect. Immun. 86, e00522–17. 10.1128/IAI.00522-17 28993461 PMC5736806

[B4] Arriola Benitez P. C. Rey Serantes D. Herrmann C. K. Pesce Viglietti A. I. Vanzulli S. Giambartolomei G. H.. (2016). The effector protein BPE005 from *Brucella abortus* induces collagen deposition and matrix metalloproteinase 9 downmodulation via transforming growth factor beta1 in hepatic stellate cells. Infect. Immun. 84, 598–606. 10.1128/IAI.01227-15 26667834 PMC4730569

[B5] Arriola Benitez P. C. Scian R. Comerci D. J. Serantes D. R. Vanzulli S. Fossati C. A.. (2013). *Brucella abortus* induces collagen deposition and MMP-9 down-modulation in hepatic stellate cells via TGF-beta1 production. Am. J. Pathol. 183, 1918–1927. 10.1016/j.ajpath.2013.08.006 24113459

[B6] Barrionuevo P. Giambartolomei G. H. (2019). Inhibition of antigen presentation by *Brucella*: many more than many ways. Microbes Infect. 21, 136–142. 10.1016/j.micinf.2018.12.004 30677519

[B7] Bertolino P. Trescol-Biémont M. C. Rabourdin-Combe C. (1998). Hepatocytes induce functional activation of naive CD8+ T lymphocytes but fail to promote survival. Eur. J. Immunol. 28, 221–36. 9485202 10.1002/(SICI)1521-4141(199801)28:01<221::AID-IMMU221>3.0.CO;2-F

[B8] Campos M. A. Rosinha G. M. Almeida I. C. Salgueiro X. S. Jarvis B. W. Splitter G. A.. (2004). Role of Toll-like receptor 4 in induction of cell-mediated immunity and resistance to *Brucella abortus* infection in mice. Infect. Immun. 72, 176–186. 10.1128/IAI.72.1.176-186.2004 14688095 PMC344000

[B9] Campos P. C. Gomes M. T. Guimarães E. S. Guimarães G. Oliveira S. C. (2017). TLR7 and TLR3 sense *Brucella abortus* RNA to induce proinflammatory cytokine production but they are dispensable for host control of infection. Front. Immunol. 8:28. 10.3389/fimmu.2017.00028 28167945 PMC5253617

[B10] Chuah C. Jones M. K. Burke M. L. McManus D. P. Gobert G. N. (2014). Cellular and chemokine-mediated regulation in schistosome-induced hepatic pathology. Trends Parasitol. 30, 141–150. 10.1016/j.pt.2013.12.009 24433721

[B11] Colmenero Jde D. Queipo-Ortuño M. I. Maria Reguera J. Angel Suarez-Muñoz M. Martín-Carballino S. Morata P. (2002). Chronic hepatosplenic abscesses in Brucellosis. Clinico-therapeutic features and molecular diagnostic approach. Diagn. Microbiol. Infect. Dis. 42, 159–167. 10.1016/S0732-8893(01)00344-3 11929686

[B12] Colmenero J. D. Reguera J. M. Martos F. Sánchez-De-Mora D. Delgado M. Causse M.. (1996). Complications associated with *Brucella melitensis* infection: a study of 530 cases. Medicine 75, 195–211. 10.1097/00005792-199607000-00003 8699960

[B13] Comerci D. J. Martínez-Lorenzo M. J. Sieira R. Gorvel J. P. Ugalde R. A. (2001). Essential role of the VirB machinery in the maturation of the *Brucella abortus*-containing vacuole. Cell. Microbiol. 3, 159–168. 10.1046/j.1462-5822.2001.00102.x 11260139

[B14] Copin R. Vitry M. A. Hanot Mambres D. Machelart A. De Trez C. Vanderwinden J. M.. (2012). *In situ* microscopy analysis reveals local innate immune response developed around *Brucella* infected cells in resistant and susceptible mice. PLoS Pathog. 8:e1002575. 10.1371/journal.ppat.1002575 22479178 PMC3315488

[B15] Corsetti P. P. de Almeida L. A. Carvalho N. B. Azevedo V. Silva T. M. Teixeira H. C.. (2013). Lack of endogenous IL-10 enhances production of proinflammatory cytokines and leads to *Brucella abortus* clearance in mice. PLoS ONE 8:e74729. 10.1371/journal.pone.0074729 24069337 PMC3775771

[B16] Costa J. D. Nogueira de Melo A. C. Vermelho A. B. Meirelles Mde N. Porrozzi R. (2008). *In vitro* evidence for metallopeptidase participation in hepatocyte damage induced by Leishmania chagasi-infected macrophages. Acta Trop. 106, 175–183. 10.1016/j.actatropica.2008.03.006 18433728

[B17] Crispe I. N. (2009). The liver as a lymphoid organ. Annu. Rev. Immunol. 27, 147–163. 10.1146/annurev.immunol.021908.132629 19302037

[B18] Cross A. R. Baldwin V. M. Roy S. Essex-Lopresti A. E. Prior J. L. Harmer N. J. (2019). Zoonoses under our noses. Microbes Infect. 21, 10–19. 10.1016/j.micinf.2018.06.001 29913297 PMC6386771

[B19] de Almeida L. A. Carvalho N. B. Oliveira F. S. Lacerda T. L. Vasconcelos A. C. Nogueira L.. (2011). MyD88 and STING signaling pathways are required for IRF3-mediated IFN-beta induction in response to *Brucella abortus* infection. PLoS ONE 6:e23135. 10.1371/journal.pone.0023135 21829705 PMC3149075

[B20] de Almeida L. A. Macedo G. C. Marinho F. A. Gomes M. T. Corsetti P. P. Silva A. M.. (2013). Toll-like receptor 6 plays an important role in host innate resistance to *Brucella abortus* infection in mice. Infect. Immun. 81, 1654–1662. 10.1128/IAI.01356-12 23460520 PMC3647997

[B21] de Figueiredo P. Ficht T. A. Rice-Ficht A. Rossetti C. A. Adams L. G. (2015). Pathogenesis and immunobiology of brucellosis: review of Brucella-host interactions. Am. J. Pathol. 185, 1505–1517. 10.1016/j.ajpath.2015.03.003 25892682 PMC4450313

[B22] Delpino M. V. Barrionuevo P. Macedo G. C. Oliveira S. C. Genaro S. D. Scian R.. (2012). Macrophage-elicited osteoclastogenesis in response to *Brucella abortus* infection requires TLR2/MyD88-dependent TNF-alpha production. J. Leukoc. Biol. 91, 285–298. 10.1189/jlb.04111185 22075930

[B23] Delpino M. V. Barrionuevo P. Scian R. Fossati C. A. Baldi P. C. (2010). *Brucella*-infected hepatocytes mediate potentially tissue-damaging immune responses. J. Hepatol. 53, 145–154. 10.1016/j.jhep.2010.02.028 20452697

[B24] Dooley S. ten Dijke P. (2012). TGF-beta in progression of liver disease. Cell Tissue Res. 347, 245–256. 10.1007/s00441-011-1246-y 22006249 PMC3250614

[B25] Eisenberg T. Hamann H. P. Kaim U. Schlez K. Seeger H. Schauerte N.. (2012). Isolation of potentially novel *Brucella* spp. from frogs. Appl. Environ. Microbiol. 78, 3753–3755. 10.1128/AEM.07509-11 22407680 PMC3346351

[B26] Fischer D. Lorenz N. Heuser W. Kämpfer P. Scholz H. C. Lierz M. (2012). Abscesses associated with a *Brucella* inopinata-like bacterium in a big-eyed tree frog (*Leptopelis vermiculatus*). J. Zoo Wildl. Med. 43, 625–628. 10.1638/2011-0005R2.1 23082529

[B27] Franco I. S. Shuman H. A. Charpentier X. (2009). The perplexing functions and surprising origins of Legionella pneumophila type IV secretion effectors. Cell. Microbiol. 11, 1435–1443. 10.1111/j.1462-5822.2009.01351.x 19563462

[B28] Freitas-Lopes M. A. Mafra K. David B. A. Carvalho-Gontijo R. Menezes G. B. (2017). Differential location and distribution of hepatic immune cells. Cells 6:E48. 10.3390/cells6040048 29215603 PMC5755505

[B29] Friedman S. L. (2008). Hepatic stellate cells: protean, multifunctional, and enigmatic cells of the liver. Physiol. Rev. 88, 125–172. 10.1152/physrev.00013.2007 18195085 PMC2888531

[B30] Gabrilovich D. I. Nagaraj S. (2009). Myeloid-derived suppressor cells as regulators of the immune system. Nat. Rev. Immunol. 9, 162–174. 10.1038/nri2506 19197294 PMC2828349

[B31] Gale R. P. Sparkes R. S. Golde D. W. (1978). Bone marrow origin of hepatic macrophages (Kupffer cells) in humans. Science 201, 937–938. 10.1126/science.356266 356266

[B32] García Samartino C. Delpino M. V. Pott Godoy C. Di Genaro M. S. Pasquevich K. A. Zwerdling A.. (2010). *Brucella abortus* induces the secretion of proinflammatory mediators from glial cells leading to astrocyte apoptosis. Am. J. Pathol. 176, 1323–1338. 10.2353/ajpath.2010.090503 20093491 PMC2830821

[B33] Giambartolomei G. H. Zwerdling A. Cassataro J. Bruno L. Fossati C. A. Philipp M. T. (2004). Lipoproteins, not lipopolysaccharide, are the key mediators of the proinflammatory response elicited by heat-killed *Brucella abortus*. J. Immunol. 173, 4635–4642. 10.4049/jimmunol.173.7.4635 15383598

[B34] Godfroid J. Al Dahouk S. Pappas G. Roth F. Matope G. Muma J.. (2013). A “One Health” surveillance and control of brucellosis in developing countries: moving away from improvisation. Comp. Immunol. Microbiol. Infect. Dis. 36, 241–248. 10.1016/j.cimid.2012.09.001 23044181

[B35] Golden-Mason L. Kelly A. M. Doherty D. G. Traynor O. McEntee G. Kelly J.. (2004). Hepatic interleuklin 15 (IL-15) expression: implications for local NK/NKT cell homeostasis and development. Clin. Exp. Immunol. 138, 94–101. 10.1111/j.1365-2249.2004.02586.x 15373910 PMC1809196

[B36] Gomes M. T. Campos P. C. Oliveira F. S. Corsetti P. P. Bortoluci K. R. Cunha L. D.. (2013). Critical role of ASC inflammasomes and bacterial type IV secretion system in caspase-1 activation and host innate resistance to *Brucella abortus* infection. J. Immunol. 190, 3629–3638. 10.4049/jimmunol.1202817 23460746

[B37] Hernandez-Gea V. Friedman S. L. (2011). Pathogenesis of liver fibrosis. Annu. Rev. Pathol. 6, 425–456. 10.1146/annurev-pathol-011110-130246 21073339

[B38] Heydtmann M. Adams D. H. (2009). Chemokines in the immunopathogenesis of hepatitis C infection. Hepatology 49, 676–688. 10.1002/hep.22763 19177577 PMC2919201

[B39] Heymann F. Tacke F. (2016). Immunology in the liver–from homeostasis to disease. Nat. Rev. Gastroenterol. Hepatol. 13, 88–110. 10.1038/nrgastro.2015.200 26758786

[B40] Hull N. C. Schumaker B. A. (2018). Comparisons of brucellosis between human and veterinary medicine. Infect. Ecol. Epidemiol. 8:1500846. 10.1080/20008686.2018.1500846 30083304 PMC6063340

[B41] Hunt A. C. Bothwell P. W. (1967). Histological findings in human brucellosis. J. Clin. Pathol. 20, 267–272. 10.1136/jcp.20.3.267 5632572 PMC473484

[B42] Kelly A. M. Golden-Mason L. Traynor O. Geoghegan J. McEntee G. Hegarty J. E.. (2006). Changes in hepatic immunoregulatory cytokines in patients with metastatic colorectal carcinoma: implications for hepatic anti-tumour immunity. Cytokine 35, 171–179. 10.1016/j.cyto.2006.07.019 16971136

[B43] Kmieć Z. (2001). Cooperation of liver cells in health and disease. Adv. Anat. Embryol. Cell Biol/ 161:III–XIII, 1-151. 10.1007/978-3-642-56553-3 11729749

[B44] Knolle P. A. Schmitt E. Jin S. Germann T. Duchmann R. Hegenbarth S.. (1999). Induction of cytokine production in naive CD4(+) T cells by antigen-presenting murine liver sinusoidal endothelial cells but failure to induce differentiation toward Th1 cells. Gastroenterology 116, 1428–1440. 10.1016/S0016-5085(99)70508-1 10348827

[B45] Kudo S. Matsuno K. Ezaki T. Ogawa M. (1997). A novel migration pathway for rat dendritic cells from the blood: hepatic sinusoids-lymph translocation. J. Exp. Med. 185, 777–784. 10.1084/jem.185.4.777 9034155 PMC2311511

[B46] Lau A. H. Thomson A. W. (2003). Dendritic cells and immune regulation in the liver. Gut 52, 307–314. 10.1136/gut.52.2.307 12524419 PMC1774973

[B47] Lohse A. W. Knolle P. A. Bilo K. Uhrig A. Waldmann C. Ibe M.. (1996). Antigen-presenting function and B7 expression of murine sinusoidal endothelial cells and Kupffer cells. Gastroenterology 110, 1175–1181. 10.1053/gast.1996.v110.pm8613007 8613007

[B48] Lopez-Sanchez I. Dunkel Y. Roh Y. S. Mittal Y. De Minicis S. Muranyi A.. (2014). GIV/Girdin is a central hub for profibrogenic signalling networks during liver fibrosis. Nat. Commun. 5:4451. 10.1038/ncomms5451 25043713 PMC4107319

[B49] Madkour M. M. (2001). Gastrointestinal brucellosis, in Madkour's Brucellosis, 2nd ed, eds Madkour M. M (Berlin: Springer-Verlag), 150–158. 10.1007/978-3-642-59533-2_13

[B50] Mancini F. R. Bella A. Graziani C. Marianelli C. Mughini-Gras L. Pasquali P.. (2014). Trends of human brucellosis in Italy, 1998-2010. Epidemiol. Infect. 142, 1188–1195. 10.1017/S0950268813002227 24044411 PMC9151243

[B51] Mao Y. Q. Fan X. M. (2015). Autophagy: a new therapeutic target for liver fibrosis. World J. Hepatol. 7, 1982–1986. 10.4254/wjh.v7.i16.1982 26261688 PMC4528272

[B52] Marchesini M. I. Herrmann C. K. Salcedo S. P. Gorvel J. P. Comerci D. J. (2011). In search of *Brucella abortus* type IV secretion substrates: screening and identification of four proteins translocated into host cells through VirB system. Cell. Microbiol. 13, 1261–1274. 10.1111/j.1462-5822.2011.01618.x 21707904 PMC3139020

[B53] Matsuno K. Ezaki T. Kudo S. Uehara Y. (1996). A life stage of particle-laden rat dendritic cells *in vivo*: their terminal division, active phagocytosis, and translocation from the liver to the draining lymph. J. Exp. Med. 183, 1865–1878. 10.1084/jem.183.4.1865 8666943 PMC2192479

[B54] McCord A. M. Resto-Ruiz S. I. Anderson B. E. (2006). Autocrine role for interleukin-8 in Bartonella henselae-induced angiogenesis. Infect. Immun. 74, 5185–5190. 10.1128/IAI.00622-06 16926411 PMC1594831

[B55] Moreno E. Moriyon I. (2002). *Brucella melitensis*: a nasty bug with hidden credentials for virulence. Proc. Natl. Acad. Sci. U.S.A. 99, 1–3. 10.1073/pnas.022622699 11782541 PMC117501

[B56] Mühlbauer M. Fleck M. Schütz C. Weiss T. Froh M. Blank C.. (2006). PD-L1 is induced in hepatocytes by viral infection and by interferon-alpha and -gamma and mediates T cell apoptosis. J. Hepatol. 45, 520–528. 10.1016/j.jhep.2006.05.007 16876901

[B57] Nemeth E. Baird A. W. O'Farrelly C. (2009). Microanatomy of the liver immune system. Semin. Immunopathol. 31, 333–343. 10.1007/s00281-009-0173-4 19639317

[B58] Ninio S. Roy C. R. (2007). Effector proteins translocated by Legionella pneumophila: strength in numbers. Trends Microbiol. 15, 372–380. 10.1016/j.tim.2007.06.006 17632005

[B59] O'Callaghan D. Cazevieille C. Allardet-Servent A. Boschiroli M. L. Bourg G. Foulongne V.. (1999). A homologue of the Agrobacterium tumefaciens VirB and Bordetella pertussis Ptl type IV secretion systems is essential for intracellular survival of Brucella suis. Mol. Microbiol. 33, 1210–1220. 10.1046/j.1365-2958.1999.01569.x 10510235

[B60] O'Farrelly C. Crispe I. N. (1999). Prometheus through the looking glass: reflections on the hepatic immune system. Immunol. Today 20, 394–398. 10.1016/S0167-5699(99)01518-2 10532788

[B61] Olsen S. C. Palmer M. V. (2014). Advancement of knowledge of Brucella over the past 50 years. Vet. Pathol. 51, 1076–1089. 10.1177/0300985814540545 24981716

[B62] Pappas G. Papadimitriou P. Akritidis N. Christou L. Tsianos E. V. (2006). The new global map of human brucellosis. Lancet Infect. Dis. 6, 91–99. 10.1016/S1473-3099(06)70382-6 16439329

[B63] Petrasek J. Bala S. Csak T. Lippai D. Kodys K. Menashy V.. (2012). IL-1 receptor antagonist ameliorates inflammasome-dependent alcoholic steatohepatitis in mice. J. Clin. Invest. 122, 3476–3489. 10.1172/JCI60777 22945633 PMC3461900

[B64] Prickett T. C. McKenzie J. L. Hart D. N. (1988). Characterization of interstitial dendritic cells in human liver. Transplantation 46, 754–761. 10.1097/00007890-198811000-00024 3057697

[B65] Puche J. E. Lee Y. A. Jiao J. Aloman C. Fiel M. I. Muñoz U.. (2013). A novel murine model to deplete hepatic stellate cells uncovers their role in amplifying liver damage in mice. Hepatology 57, 339–350. 10.1002/hep.26053 22961591 PMC3522764

[B66] Robinson M. W. Harmon C. O'Farrelly C. (2016). Liver immunology and its role in inflammation and homeostasis. Cell. Mol. Immunol. 13, 267–276. 10.1038/cmi.2016.3 27063467 PMC4856809

[B67] Rolán H. G. Tsolis R. M. (2008). Inactivation of the type IV secretion system reduces the Th1 polarization of the immune response to *Brucella abortus* infection. Infect. Immun. 76, 3207–3213. 10.1128/IAI.00203-08 18458071 PMC2446720

[B68] Roux C. M. Rolán H. G. Santos R. L. Beremand P. D. Thomas T. L. Adams L. G.. (2007). Brucella requires a functional Type IV secretion system to elicit innate immune responses in mice. Cell. Microbiol. 9, 1851–1869. 10.1111/j.1462-5822.2007.00922.x 17441987

[B69] Sander L. E. Sackett S. D. Dierssen U. Beraza N. Linke R. P. Müller M.. (2010). Hepatic acute-phase proteins control innate immune responses during infection by promoting myeloid-derived suppressor cell function. J. Exp. Med. 207, 1453–1464. 10.1084/jem.20091474 20530204 PMC2901069

[B70] Santos S. A. Andrade D. R. Andrade Júnior D. R. (2005). Rat hepatocyte invasion by Listeria monocytogenes and analysis of TNF-alpha role in apoptosis. Rev. Inst. Med. Trop. São Paulo 47, 73–80. 10.1590/S0036-46652005000200003 15880217

[B71] Sieira R. Comerci D. J. Sánchez D. O. Ugalde R. A. (2000). A homologue of an operon required for DNA transfer in Agrobacterium is required in *Brucella abortus* for virulence and intracellular multiplication. J. Bacteriol. 182, 4849–4855. 10.1128/JB.182.17.4849-4855.2000 10940027 PMC111363

[B72] Smith K. (2013). Liver disease: kupffer cells regulate the progression of ALD and NAFLD. Nat. Rev. Gastroenterol. Hepatol. 10:503. 10.1038/nrgastro.2013.140 23877532

[B73] Song Y. Zhao Y. Wang F. Tao L. Xiao J. Yang C. (2014). Autophagy in hepatic fibrosis. Biomed Res. Int. 2014:436242. 10.1155/2014/436242 24779010 PMC3980865

[B74] Steffan A. M. Gendrault J. L. McCuskey R. S. McCuskey P. A. Kirn A. (1986). Phagocytosis, an unrecognized property of murine endothelial liver cells. Hepatology 6, 830–836. 10.1002/hep.1840060505 3758936

[B75] Villar J. M. Garrote D. Villegas M. T. Alvarez M. J. Mansilla A. Ferrón J. A. (2002). Hepatic brucelloma. J. Am. Coll. Surg. 194:86. 10.1016/S1072-7515(01)01111-5 11800344

[B76] Xavier M. N. Winter M. G. Spees A. M. Nguyen K. Atluri V. L. Silva T. M.. (2013). CD4+ T cell-derived IL-10 promotes *Brucella abortus* persistence via modulation of macrophage function. PLoS Pathog. 9:e1003454. 10.1371/journal.ppat.1003454 23818855 PMC3688575

[B77] Xu R. Zhang Z. Wang F. S. (2012). Liver fibrosis: mechanisms of immune-mediated liver injury. Cell. Mol. Immunol. 9, 296–301. 10.1038/cmi.2011.53 22157623 PMC4132585

[B78] Yin X. M. Ding W. X. Gao W. (2008). Autophagy in the liver. Hepatology 47, 1773–1785. 10.1002/hep.22146 18393362

[B79] Young E. J. (1995). An overview of human brucellosis. Clin. Infect. Dis. 21, 283–9; quiz 90. 10.1093/clinids/21.2.283 8562733

[B80] Zechner E. L. Lang S. Schildbach J. F. (2012). Assembly and mechanisms of bacterial type IV secretion machines. Philos. Trans. R. Soc. Lond,. B,. Biol. Sci. 367, 1073–1087. 10.1098/rstb.2011.0207 22411979 PMC3297438

[B81] Zimmermann H. W. Bruns T. Weston C. J. Curbishley S. M. Liaskou E. Li K. K.. (2015). Bidirectional transendothelial migration of monocytes across hepatic sinusoidal endothelium shapes monocyte differentiation and regulates the balance between immunity and tolerance in liver. Hepatology 63, 233–246. 10.1002/hep.28285 26473398 PMC6016741

[B82] Zwerdling A. Delpino M. V. Barrionuevo P. Cassataro J. Pasquevich K. A. García Samartino C.. (2008). Brucella lipoproteins mimic dendritic cell maturation induced by *Brucella abortus*. Microbes Infect. 10, 1346–1354. 10.1016/j.micinf.2008.07.035 18761420

[B83] Zwerdling A. Delpino M. V. Pasquevich K. A. Barrionuevo P. Cassataro J. García Samartino C.. (2009). *Brucella abortus* activates human neutrophils. Microbes Infect. 11, 689–697. 10.1016/j.micinf.2009.04.010 19376263

